# Novel in situ seeding immunodetection assay uncovers neuronal-driven alpha-synuclein seeding in Parkinson’s disease

**DOI:** 10.1038/s41531-025-01111-y

**Published:** 2025-08-25

**Authors:** Maria Otero-Jimenez, Marcelina J. Wojewska, Simona Jogaudaite, David Miller, Sandra Gray-Rodriguez, Grainne C. Geoghegan, Laura Abelleira-Hervas, Tim James Viney, Barbara Sarkany, Djordje Gveric, Steve Gentleman, Javier Alegre-Abarrategui

**Affiliations:** 1 Department of Brain Sciences, Imperial College London, Hammersmith Hospital, London, UK; 2 Imperial College Advanced Hackspace, Imperial College London, London, UK; 3 Department of Pharmacology, University of Oxford, Oxford, UK

**Keywords:** Biological techniques, Biotechnology, Neuroscience, Biomarkers, Diseases, Medical research, Neurology, Pathogenesis

## Abstract

Aggregates of alpha-synuclein (α-syn) propagate through template-induced misfolding in people with Parkinson’s disease (PD) and Multiple System Atrophy (MSA). Prion-like seeding is crucial in disease initiation and progression, representing a major target for disease-modifying therapies. The detection of α-syn seeding with seeding amplification assays (SAAs) has remarkable diagnostic and research potential. However, current SAAs rely on bulk tissue homogenates or fluids, losing critical spatial and cellular resolution. Here, we report our novel in situ seeding immunodetection (*is*SID) assay that enables the visualization of seeding with unprecedented morphological detail in intact biological tissue. Using the *is*SID assay, we confirm seeding activity in α-syn aggregates in PD, MSA, and other proteinopathies, while uncovering neuron-driven seeding preceding the clinical symptom onset in PD. Our findings provide new fundamental insights into the pathogenesis underlying neurodegeneration and establish an invaluable tool for studying protein aggregation dynamics, with potential applications in biomarker discovery, diagnostics, and therapeutics.

## Introduction

Many common neurodegenerative disorders are characterized by the aggregation of misfolded proteins. These form pathological inclusions^1,2^, disrupting cellular function and eventually leading to neuronal cell death^2^.

Alpha-synucleinopathies are a group of neurodegenerative disorders characterized by the aggregation of alpha-synuclein (α-syn), a protein thought to play a physiological role in synaptic function^3^. These diseases include Parkinson’s disease (PD), Parkinson’s Disease Dementia (PDD), Dementia with Lewy Bodies (DLB), and Multiple System Atrophy (MSA). The defining neuropathological feature of PD, PDD, and DLB, collectively termed “Lewy body disease” (LBD), is the presence of α-syn aggregates within neurons forming Lewy bodies (LBs) and Lewy neurites (LNs), which can be visualized histologically. However, the presence of glial pathology is becoming increasingly recognized. Recently, we reported a distinct pattern of neuroanatomical pathological progression, in which neurons are the first affected cell type, while the involvement of astrocytes only occurs when α-syn pathology becomes widespread^4^. In contrast, MSA is characterized by α-syn aggregates within oligodendrocytes, manifesting as glial cytoplasmic inclusions (GCIs) in addition to a lower prevalence of neuronal cytoplasmic inclusions (NCIs) and neuronal nuclear inclusions (NNIs)^5,6^. Additionally, 8–17% of aged neurologically healthy individuals exhibit LBs upon *post-mortem* examination, a phenomenon termed incidental Lewy body disease (iLBD), which is thought to represent a prodromal stage of PD^7^.

While the precise factors driving protein misfolding and aggregation remain incompletely understood, evidence suggests that pathological aggregates of misfolded proteins, such as α-syn, can propagate in a prion-like manner^8^. This mechanism is hypothesized to be shared by other protein-misfolding diseases like tauopathies, which are characterized by the aggregation of hyperphosphorylated tau^9–11^. Prion-like seeding involves the aberrant misfolding of native proteins by pathological aggregates. During this process, oligomeric species and fibrillar inclusions are formed, facilitating disease initiation and progression^12^. However, whether all pathological forms present across cell types are equally seeding-competent remains to be elucidated.

The prion-like activity of α-syn, tau, and other amyloidogenic proteins has been exploited to develop both cell-based and cell-free seeding assays. Cell-based assays commonly utilize biosensor cell lines, such as HEK293T cell lines stably expressing α-syn or tau fused to cyan fluorescent protein (CFP) and yellow fluorescent protein (YFP). Liposome-mediated transduction of proteopathic seeds, either patient-derived or pre-formed fibrils (PFFs), into biosensor cells induces aggregation of CFP- and YFP-tagged proteins, generating fluorescence resonance energy transfer (FRET) signals and the formation of cytoplasmic inclusions^13,14^. Cell-free seeding assays, such as seeding amplification assays (SAAs), include protein-misfolding cyclic amplification (PMCA) and real-time quaking-induced conversion (RT-QuIC), which were originally developed for the detection of misfolded prion proteins^15,16^. The PMCA assay involves cyclic sonication and incubation with vast excesses of normal brain homogenates containing endogenous monomers^15^. The RT-QuIC assay utilizes a reaction buffer containing recombinant monomeric protein, the fluorescent dye thioflavin T (ThT), and samples containing the protein aggregates of interest^16–18^. Although originally developed using homogenized brain tissue, the assay has since evolved to be compatible with cerebrospinal fluid (CSF), skin, olfactory mucosa, and gut tissue^19–23^. The reaction mixture is subjected to orbital shaking, and if pathological protein is present in the sample, it seeds the aggregation of the recombinant monomers. The aggregation process involves cycles of elongation and fragmentation, which are monitored in real time through the fluorescence emitted by the intercalation of ThT into the newly formed aggregates^24^. Since their development, cell-free seeding assays have been adapted to detect pathological prion-like proteins such as α-syn, tau, and TAR DNA-binding protein (TDP-43)^25,26^. Importantly, both the α-syn and tau RT-QuIC assays are able to successfully detect positive responses even in prodromal cases before the appearance of overt clinical symptoms^27–31^.

Despite its utility, the RT-QuIC assay requires bulk sample input; thus, the spatiotemporal information about the seeding activity is lost. A detailed understanding of the cellular and subcellular distribution of seeding activity is critical in proteinopathies, which are characterized by selective cellular vulnerability^32–34^. For instance, more than a century after the discovery of LBs, it remains unknown whether LBs and other types of α-syn inclusions possess seeding activity^35^. Recent studies have now demonstrated that α-syn seeding activity information can be obtained from formalin-fixed paraffin-embedded (FFPE) tissue, a widely used method to preserve and archive tissue^36–38^. Studies have confirmed seeding activity in a range of brains fixed from only 18 hours to archival tissue fixed for over 20 years^36–38^. However, the samples still required aggressive sample preparation steps, including tissue homogenization, sonication, deparaffinization, dissociation, and heat-mediated antigen retrieval. Remarkably, these studies demonstrated that the seeding activity in FFPE tissue from cases with proteinopathies was comparable to that in frozen tissue, and accordingly minimal in control samples.

Hence, as FFPE-tissue sections likely preserve seeding activity, we next sought to develop a new assay to visualize seeding activity within intact tissue. For this, we developed the novel in situ seeding immunodetection (*is*SID) assay, which leverages the endogenous seeding mechanism utilized in SAAs, combined with in situ visualization through immunostaining techniques. In this study, we describe the *is*SID assay for α-syn and tau, revealing for the first time the spatial and cellular distribution of seeding activity with fully preserved histomorphology in a cohort of PD, DLB, MSA, iLBD, and Alzheimer's disease (AD) cases. Applying this novel assay, we found that neurons are likely the primary drivers of α-syn seeding, with glial cells actively contributing to the seeding process. Additionally, we detected α-syn seeding in asymptomatic individuals with incidental α-syn pathology, demonstrating that α-syn seeding precedes the onset of clinical symptoms.

## Results

### The *is*SID assay specifically localizes endogenous α-syn and tau seeding activity in human brain tissue with intact morphological detail

**Fig. 1:**
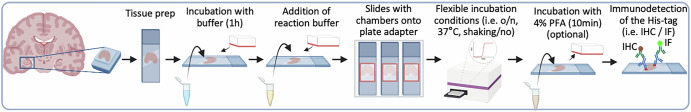
Workflow of the *is*SID assay on tissue sections. In tissue sections, slides undergo tissue preparation followed by a 1-hour incubation in the designated buffer. The reaction mixture containing the buffer, His-tagged monomeric substrate, and ThT is then applied to the tissue section. Slides are placed into a plate adapter, and the chambers are sealed. The slides are incubated under customizable conditions (i.e., shaking, time, temperature). Following this, tissue sections are fixed with 4% PFA for 10 minutes (optional) and the His-tag is detected by IHC or immunofluorescence.

**Fig. 2:**
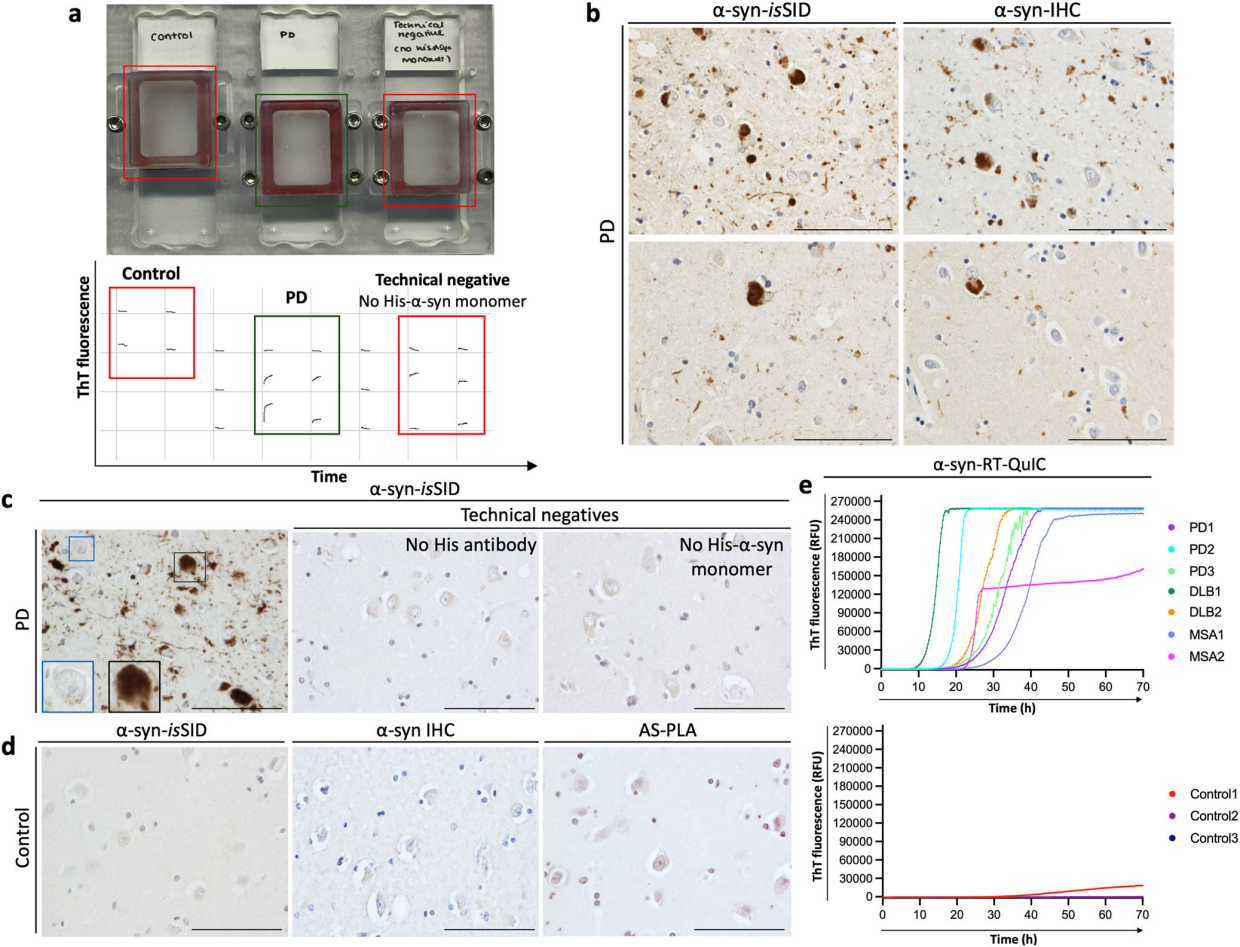
Validation of the specificity of the α-syn-*is*SID assay. Overview of the α-syn-*is*SID assay setup. A marked increase in the ThT fluorescence was detected exclusively in cases with previously characterized α-syn pathology, exemplified by a representative PD case (green box). No relative increase in ThT signal was detected in a representative control case or in the technical negative where His-α-syn monomer was not added (red boxes) (**a**). Areas of positive α-syn-*is*SID signal corresponded with areas of α-syn pathology detected by IHC in PD cases. Using amygdala sections from the same patient, α-syn-*is*SID and α-syn-IHC were performed, where a representative case with high pathology (top) and low pathology (bottom) are shown (**b**). In the amygdala of a PD case, extensive seeding-competent α-syn pathology was observed (gray square), while neurons lacking α-syn pathology (blue square) were also present, confirming signal specificity. Omission of either the anti-His antibody or recombinant His-α-syn substrate during incubation abolished signal, confirming that the assay specifically detects newly aggregated monomeric His-α-syn in situ (**c**). Control cases without α-syn pathology, as confirmed by α-syn-IHC and AS-PLA, showed no signal in α-syn-*is*SID (**d**). RT-QuIC curves confirm consistency with α-syn-*is*SID results. Positive RT-QuIC responses were observed in PD, DLB, and MSA cases with α-syn-*is*SID signal, while control cases lacking both α-syn pathology and α-syn-*is*SID signal showed negative RT-QuIC responses. RT-QuIC traces represent averaged triplicate samples, and fluorescence is shown in relative fluorescence units (RFU) (**e**). Scale bar is 100 µm; insets show magnified regions.

To understand cell type-specific α-syn seeding activity in a spatial context, we developed the α-syn-*is*SID assay. We first explored whether FFPE-tissue sections had seeding-competent α-syn that could be visualized in situ. For this, tissue sections were subjected to our novel *is*SID assay, in which the tissue sections were incubated with His-α-syn substrate (Fig. 1). Tissue with α-syn pathology, as confirmed by both α-syn immunohistochemistry (IHC) and proximity ligation assay (PLA), exhibited an increase of ThT fluorescence, which was used as an intra-assay monitoring measure, while sections without α-syn pathology or incubated without His-α-syn monomers had no increase in ThT fluorescence (Fig. 2a). Then, we attempted to visualize nascent α-syn aggregates seeded in situ using IHC with antibodies against the His-tag, demonstrating that cases with α-syn pathology also displayed positive signal in the α-syn-*is*SID assay (Fig. 2b). Signal was also abolished when either the His-α-syn monomeric substrate or the anti-His antibody was omitted, confirming that the detected signal results from de novo aggregation of exogenous His-α-syn monomer seeded in situ by endogenous α-syn aggregates, and does not represent spontaneous aggregation (Fig. 2c). Since the α-syn-*is*SID signal was solely observed in the presence of pathological α-syn, no signal was detected in any control cases lacking α-syn pathology, yielding no false positives (Fig. 2d). Importantly, all α-syn-positive cases, regardless of the diagnosis (PD, DLB, or MSA), consistently showed α-syn-*is*SID signal that corresponded with conventional RT-QuIC results performed on frozen brain homogenates from the same regions (Fig. 2e). Similarly, control cases with no α-syn pathology yield no RT-QuIC signal, highlighting the strong concordance between the assays and the added spatial information provided by the α-syn-*is*SID assay.

**Fig. 3:**
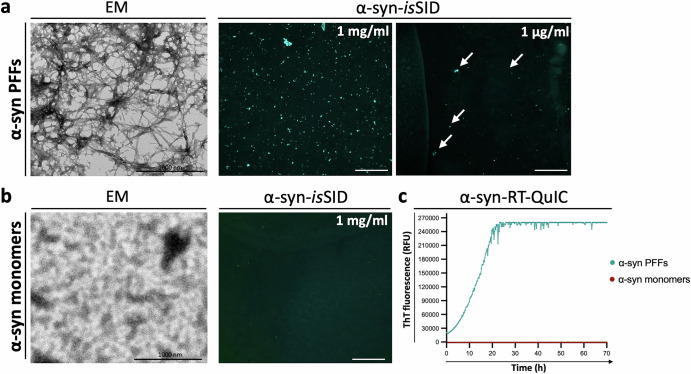
Validation of the sensitivity of the α-syn-*is*SID assay. Representative images of independent experiments are shown. Electron microscopy (EM) analysis of α-syn PFFs (left). When α-syn-*is*SID is assayed on poly-D-lysine-coated coverslips spotted with α-syn PFFs, robust fluorescent signal is detected at 1 mg/mL, with reduced but still detectable signal at 1 µg/mL (right), representing the assay’s sensitivity (**a**). EM analysis of α-syn monomers (left). Correspondingly, no signal is observed when α-syn-*is*SID is applied to α-syn monomers (right), supporting the assay’s specificity for aggregated species (**b**). RT-QuIC assay confirms the seeding competence of α-syn PFFs and the lack of seeding activity of monomeric α-syn (**c**). Scale bar of the EM images is 1000 nm, and 100 µm for the α-syn-*is*SID images.

To evaluate the detection sensitivity and dynamic range of the α-syn-*is*SID assay, we applied it to serial dilutions of α-syn PFFs spotted onto coated coverslips. The α-syn-*is*SID assay consistently detected seeding in α-syn PFFs across a wide concentration range, from 1 mg/mL down to 1 µg/mL, corresponding to a detection threshold of at least ~5 ng of α-syn PFFs per droplet. No signal was observed in the absence of His-α-syn substrate or when only monomeric α-syn was applied, indicating the high specificity of the α-syn-*is*SID assay at detecting seeding activity in aggregated α-syn species (Fig. 3; Supplementary Fig. 1).

We then wanted to investigate if α-syn-*is*SID could be adapted to investigate seeding activity using other α-syn protein substrates, such as the A53T mutant, instead of the wild-type (WT) His-α-syn substrate^2,39^. The A53T α-syn mutant protein was successfully and effectively shown to be used to explore α-syn seeding in situ, revealing *is*SID signal in LBs, astrocytes, and oligodendrocytes (Supplementary Fig. 2).

**Fig. 4:**
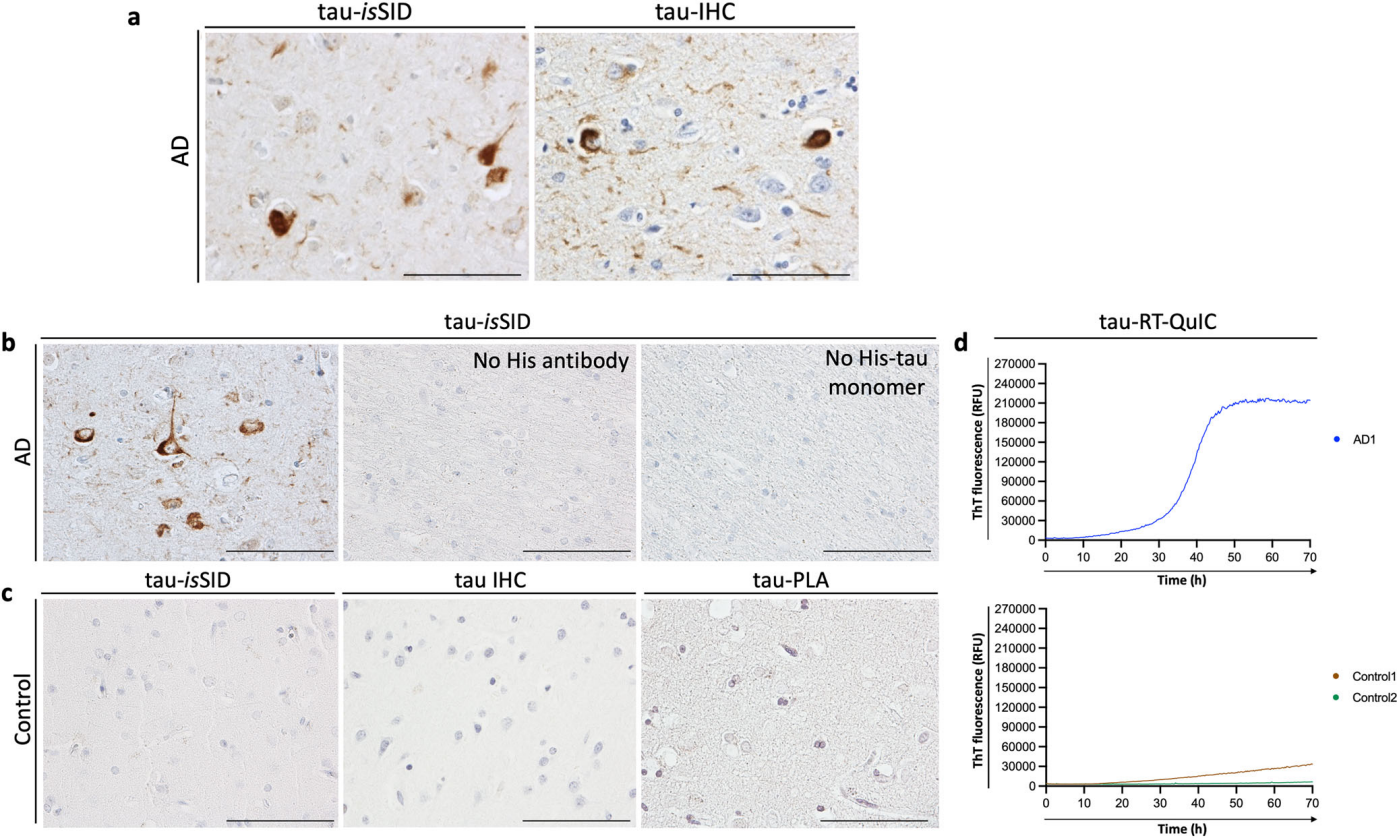
Validation of the tau-*is*SID specificity. Tau-*is*SID signal was detected in the temporal cortex of an AD case with previously characterized tau pathology as detected by tau-IHC (**a**). Notably, omission of either the anti-His antibody or monomeric His-tau substrate during incubation resulted in loss of signal, demonstrating that the assay specifically detects newly aggregated recombinant His-tau in situ (**b**). Control cases without detectable tau pathology, as confirmed by either tau-IHC or tau-PLA, displayed no tau-*is*SID signal (**c**). A positive RT-QuIC response was observed in an AD case with tau-*is*SID signal, while control cases lacking tau pathology and tau-*is*SID signal showed negative RT-QuIC responses. RT-QuIC traces represent averaged triplicate samples, and fluorescence is shown in relative fluorescence units (RFU) (**d**). Scale bar is 100 µm.

While the main purpose of this study was to establish the spatial distribution of endogenous α-syn seeding activity, we also wanted to test whether this novel assay could be extended to other proteins with prion-like seeding properties. By modifying the *is*SID protocol to incorporate His-tagged 4-repeat (4 R) tau monomers as the substrate, we applied the assay to a cohort of AD cases. The tau-*is*SID assay successfully demonstrated that tau seeding activity can be visualized in situ in AD cases with tau pathology confirmed by tau-IHC (Fig. 4a). As with α-syn, tau-*is*SID produced no signal under control conditions lacking either the His-tagged monomeric protein or the anti-His antibody, confirming the assay’s specificity and ruling out non-specific background signal (Fig. 4b). Also, no detectable tau seeding activity was observed in cases without tau pathology, as defined by both tau-IHC and tau-PLA (Fig. 4c). Moreover, AD cases with confirmed tau pathology and positive tau-*is*SID signals also exhibited seeding activity in the conventional RT-QuIC assay (Fig. 4d). In contrast, control cases lacking tau pathology showed no detectable seeding activity in either RT-QuIC or tau-*is*SID, further supporting the specificity and robustness of the assay (Fig. 4d).

Shaking is a key factor required for bulk SAAs. Therefore, we sought to explore whether shaking was essential in our α-syn and tau *is*SID assays. We observed that α-syn-*is*SID was able to detect α-syn seeding activity in situ under non-shaking conditions, with results comparable to those obtained using the shaking protocol. In contrast, the tau-*is*SID assay produced significantly weaker signals under non-shaking conditions, and only dot-like foci of seeding activity in the soma of neurons and in the neuropil remained detectable (Supplementary Fig. 3). All experiments we describe in this manuscript, except where indicated, were performed under shaking conditions.

### The *is*SID assay reveals both neuronal and glial α-syn inclusions, and tau aggregates possess prion-like seeding activity

**Fig. 5:**
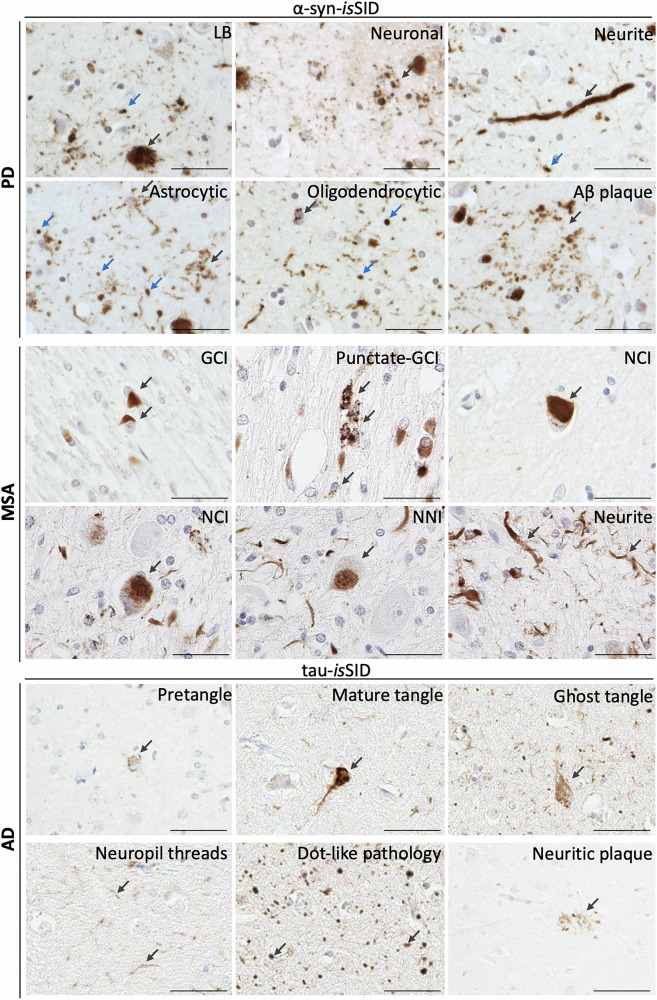
The *is*SID assay confirms that pathological α-syn and tau inclusions possess seeding potential. In PD cases, α-syn-*is*SID labeled pathological α-syn aggregates in LBs, neuronal, and neuritic inclusions, as well as in astrocytes, oligodendrocytes, and on the periphery associated with an Aβ plaque. Blue arrows indicate dot-like pathology. In MSA cases, the α-syn-*is*SID confirmed seeding capacity in GCIs, punctate GCIs, as well as neuronal inclusions, including NCIs, NNIs, and neurites. In AD cases, tau-*is*SID detects seeding potential within pathological tau structures, including pretangles, mature neurofibrillary tangles, ghost tangles, neuropil threads, dot-like pathology, and neuritic plaques. Scale bar is 50 µm.

Following validation of the specificity of the α-syn-*is*SID and tau-*is*SID assays, we investigated some unresolved questions regarding the seeding capacity of LBs and other neuronal pathologies, as well as glial α-syn aggregates. By applying α-syn-*is*SID to PD and DLB brain tissue, we observed seeding-competent α-syn pathological inclusions across several cell types. Importantly, using this novel technique, we were able to confirm that LBs are not inert. In addition, we identified seeding-competent α-syn neuronal structures with the morphology of LNs. Beyond neuronal pathology, α-syn-*is*SID revealed that α-syn glial inclusions have seeding capacity, including those in astrocytes and oligodendrocytes (Fig. 5). The seeding-competent astrocytic α-syn inclusions matched all our recently described novel morphologies^4^. To further verify the cellular identity of seeding-positive structures, we performed immunofluorescence to co-localize α-syn-*is*SID with a variety of cell markers in PD cases. These analyses confirmed that α-syn-*is*SID signal was present in neurons, astrocytes, oligodendrocytes, as well as in microglia (Supplementary Fig. 4). Furthermore, in addition to well-characterized inclusions, we detected α-syn dot-like foci of seeding activity scattered in the neuropil and in the perikaryal cytoplasm of certain neurons. These dot-like structures may represent early stages in the formation of LN and LB, respectively^40^. Neuropil dot-like seeding foci predominantly co-localized with neurofilament and synaptic markers, indicating their neuronal, and more specifically, synaptic location (Supplementary Fig. 5). Moreover, we observed a positive signal in the vicinity of neuritic amyloid-beta (Aβ)plaques (Fig. 5)^41,42^.

The α-syn-*is*SID was next applied to investigate whether α-syn aggregates in MSA had seeding activity. Our results demonstrate that GCIs present in oligodendrocytes exhibit robust seeding capacity. Interestingly, a subpopulation of oligodendrocytes displayed punctate cytoplasmic seeding activity (“punctate GCIs”), suggesting structural heterogeneity among α-syn-positive glial inclusions. Moreover, we also detected seeding-competent α-syn in neuronal inclusions, including NCIs and NNIs, alongside neurite-like structures. These findings indicate that although GCIs constitute the predominant lesion type in MSA, seeding activity is not restricted to oligodendrocytic inclusions but also to the less common neuronal α-syn pathology (Fig. 5).

Although alpha-synucleinopathies were the primary focus of this study, we also sought to investigate whether our findings with α-syn-*is*SID extended to tau pathology. In AD cases, the tau-*is*SID assay detected seeding-competent tau aggregates across a spectrum of pathological structures, including pretangles, mature neurofibrillary tangles (NFTs), ghost tangles, neuropil threads, and the dystrophic neurites. Similar to observations with α-syn, dot-like foci of tau seeding activity were observed in the neuropil and in the perikaryal cytoplasm of some neurons (Fig. 5).

To further examine the relationship between seeding activity and established α-syn and tau pathology, we stained consecutive sections of PD and AD brains with α-syn/tau-*is*SID, AS/tau-PLA, and α-syn/tau-IHC, respectively. Classical pathological aggregates of α-syn and tau, such as LBs and NFTs, were detected by all three methods. However, the dot-like neuropil foci of seeding activity observed with both α-syn- and tau-*is*SID showed greater spatial correspondence with PLA signal than with IHC, suggesting that these may represent oligomeric species displaying seeding activity (Supplementary Fig. 6).

### Neuroanatomical and cell type distribution of α-syn seeding activity in PD and in MSA

We next examined the neuroanatomical and cell type distribution of α-syn seeding activity present across various stages of PD as well as in MSA. Brain regions known to be affected at different points during disease pathogenesis were selected, namely the medulla, pons (locus coeruleus, LC), midbrain (substantia nigra, SN), cerebellum, amygdala, hippocampus, cingulate and entorhinal cortices.

**Fig. 6:**
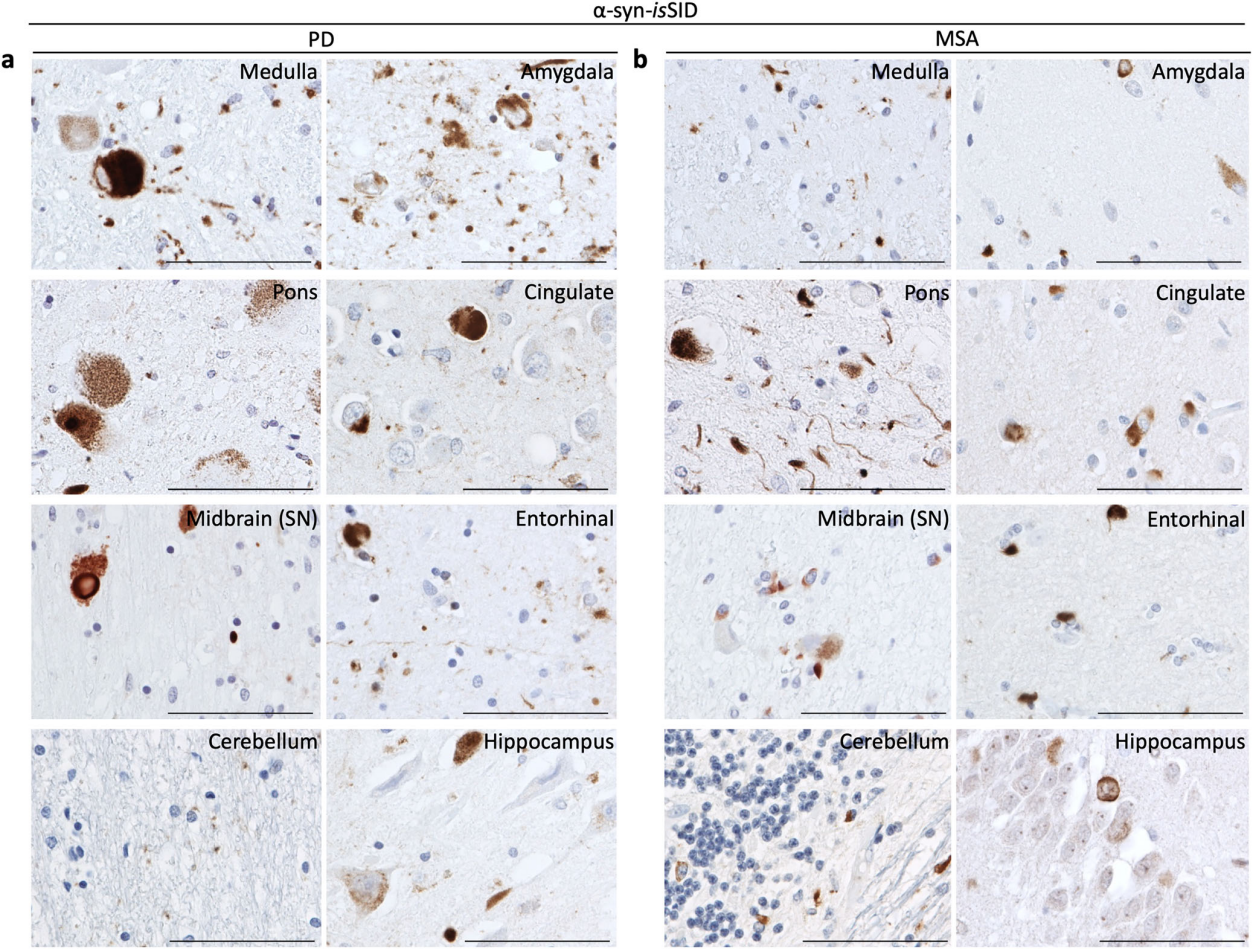
Distribution of α-syn seeding activity across distinct neuroanatomical brain regions in PD and MSA cases using the α-syn-*is*SID assay. Representative images illustrate the seeding activity assessed in multiple brain regions, including the medulla, pons, midbrain (SN), cerebellum, amygdala, cingulate cortex, entorhinal cortex, and the hippocampus using α-syn-*is*SID assay in PD (**a**) and MSA cases (**b**). Scale bar is 100 µm.

In PD, brainstem-type LBs exhibited robust seeding capacity across the medulla, LC, and SN. Notably, the characteristic halo of nigral LBs seen in dopaminergic neurons was labeled by the assay. In the medulla, both glial α-syn inclusions and α-syn-positive fiber tracts exhibited the ability to seed. The amygdala harbored a variety of α-syn-positive cellular inclusions, including LBs, neuronal inclusions, LNs, as well as glial inclusions in astrocytes and oligodendrocytes, all of which displayed seeding competence. In the cingulate and entorhinal cortices, cortical LBs alongside associated glial inclusions were also seeding-competent. In the hippocampus, widespread seeding activity was observed, particularly in neuronal populations and processes within the CA2/3 region and entorhinal cortex. Interestingly, although the cerebellar white matter is largely unaffected in PD, the α-syn-*is*SID revealed sparse “dot-like” labeling in the neuropil, suggestive of early or small oligomeric pathology (Fig. 6a).

In MSA, we observed widespread α-syn seeding activity across all assessed brain regions, including diverse morphologies of GCIs. In the medulla, both GCIs and neuronal α-syn inclusions in the form of NNIs and NCIs displayed α-syn seeding activity. The pons contained extensive α-syn-*is*SID signal, labeling neuronal inclusions and neuritic structures throughout all the transverse pontine fiber tracts. Seeding-competent neuronal inclusions were also detected in the amygdala and cortex, as well as ring-like neuronal inclusions in the dentate gyrus. In the cerebellum, α-syn seeding activity was detected in GCIs, “punctate GCIs”, and neuronal α-syn inclusions, confirming the widespread nature of α-syn pathogenesis in MSA (Fig. 6b).

### The *is*SID assay reveals increased α-syn seeding activity in neurons as compared to glia in PD

Next, we quantified the relative proportions of neurons, astrocytes, and oligodendrocytes possessing seeding-competent α-syn pathology. To do this, we compared the number of each type of cell exhibiting α-syn seeding activity, as detected by α-syn-*is*SID, to those detected by conventional α-syn-IHC. To correlate these measures, we used consecutive tissue sections from the amygdala of both PD and MSA cases.

**Fig. 7:**
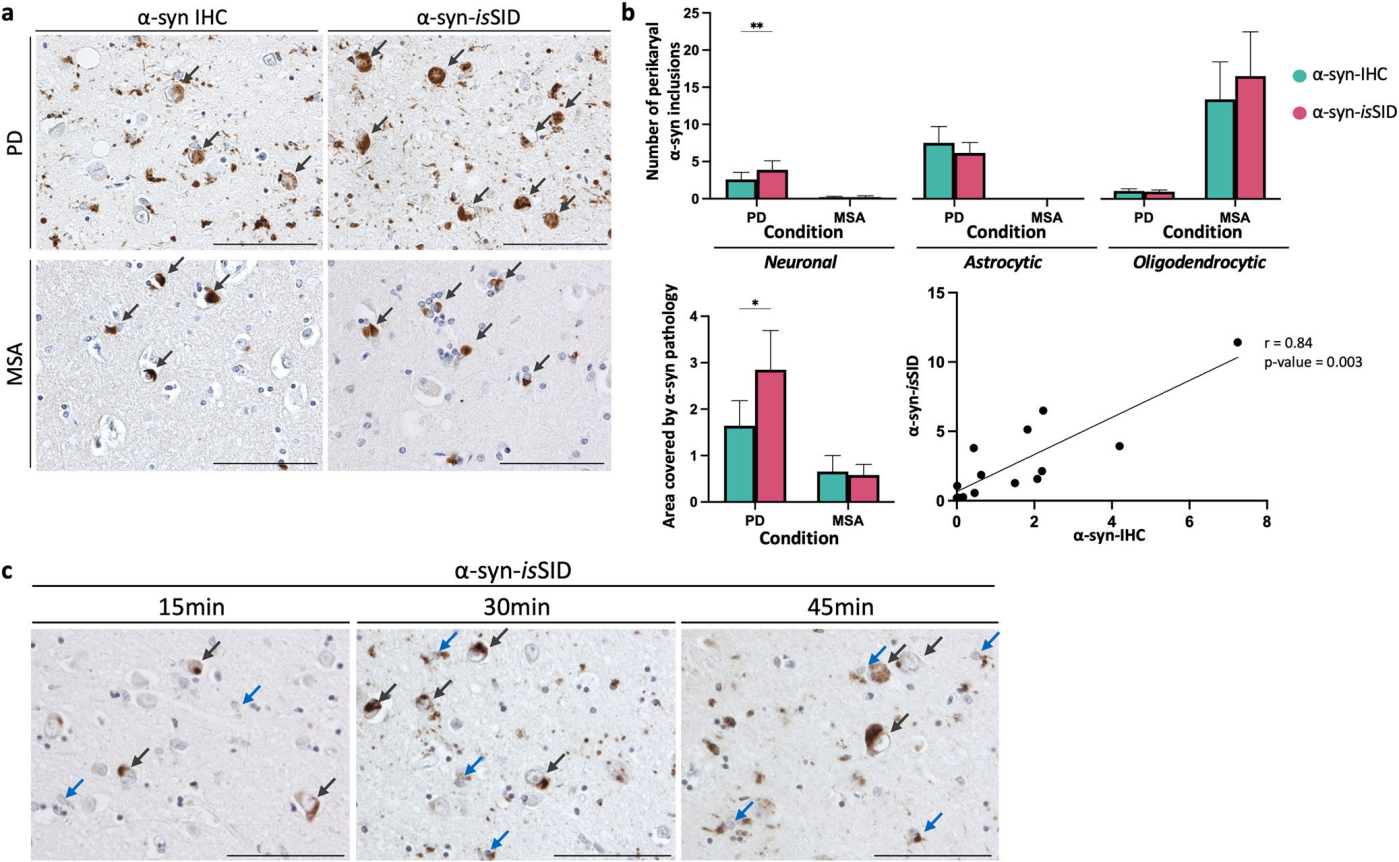
Neuronal α-syn pathology exhibits higher seeding activity than glial pathology in PD. Aggregates of α-syn and their seeding capacity were analyzed in the amygdala of PD and MSA cases using α-syn-IHC and α-syn-*is*SID. Representative images of neuronal inclusions detected by α-syn-*is*SID in PD and MSA (gray arrows) (**a**). Quantification of cell inclusions detected by each technique revealed that α-syn-*is*SID detects more neuronal pathology in PD compared to α-syn-IHC, whereas in MSA, both techniques detected similar levels of α-syn pathology. Oligodendrocytic and astrocytic α-syn pathology showed no significant differences between α-syn-IHC and α-syn-*is*SID in either PD or MSA cases. Quantification of total percentage area covered by α-syn pathology (representing α-syn burden) revealed that α-syn-*is*SID detected more α-syn pathology than α-syn-IHC in PD cases. Statistical analysis was performed using a two-way ANOVA followed by a Bonferroni *post-hoc* test (*p < 0.05; **p < 0.01). Bars represent the mean + standard error of the mean. The scatter plot illustrates a strong positive correlation between α-syn-IHC and α-syn-*is*SID signal in PD (*r* = 0.84; *p* value = 0.0003), as revealed by Spearman’s rank correlation test (**b**). Consecutive PD sections subjected to short α-syn-*is*SID incubation times displayed more prominent neuronal α-syn inclusions compared to glial inclusions at 15 minutes, although they were still detectable and increased over time. Gray arrows indicate neuronal α-syn inclusions; blue arrows indicate glial α-syn inclusions (**c**). Scale bar is 100 µm.

In PD cases, the total pathological signal detected by α-syn-*is*SID assay was higher than the α-syn pathology detected by α-syn-IHC, although the signals obtained by both methods were highly correlated (Fig. 7a, b). Importantly, significantly more neurons displayed α-syn seeding than the number of neuronal inclusions detected by α-syn-IHC, suggesting that the assay reveals early-stage or otherwise undetectable α-syn aggregates. In contrast, the number of astrocytic and oligodendrocytic α-syn inclusions did not significantly differ between the techniques. In MSA cases, α-syn-*is*SID and α-syn-IHC detected similar numbers of oligodendrocytic and neuronal α-syn inclusions. As expected, astrocytic α-syn inclusions were negligible in MSA and showed no significant difference with either technique (Fig. 7a, b).

Both neurons and glial cells displayed α-syn seeding capacity, however additional α-syn seeding-competent neurons were revealed by α-syn-*is*SID in PD cases. To further determine any differential α-syn seeding dynamics between neuronal and glial α-syn inclusions, α-syn-*is*SID was performed with a time series using sequential tissue sections of PD cases. Incubation times of 15, 30, and 45 minutes were tested to assess the temporal profile of labeling across different cell types. At 15 minutes, the incipient but widespread α-syn-*is*SID signal was predominantly observed in neurons, albeit at a reduced intensity compared to the standard overnight incubation protocol with His-α-syn. In contrast, the labeling of glial α-syn pathology showed more restricted and focal signal at this early time point. With increased incubation durations of 30 and 45 minutes, both the number of glial inclusions and the overall signal area labeled by α-syn-*is*SID increased (Fig. 7c). These findings suggest that neuronal α-syn pathology possesses increased or more rapid seeding activity than its glial counterparts.

### Seeding-competent α-syn is found in asymptomatic cases with incidental α-syn pathology

To assess the early cell type distribution of α-syn seeding activity in PD, we applied the α-syn-*is*SID assay to the brainstem of cases with Braak stage 1-3 pathology, categorized as iLBD cases. These cases, considered asymptomatic, exhibited α-syn pathology restricted to the brainstem.

**Fig. 8:**
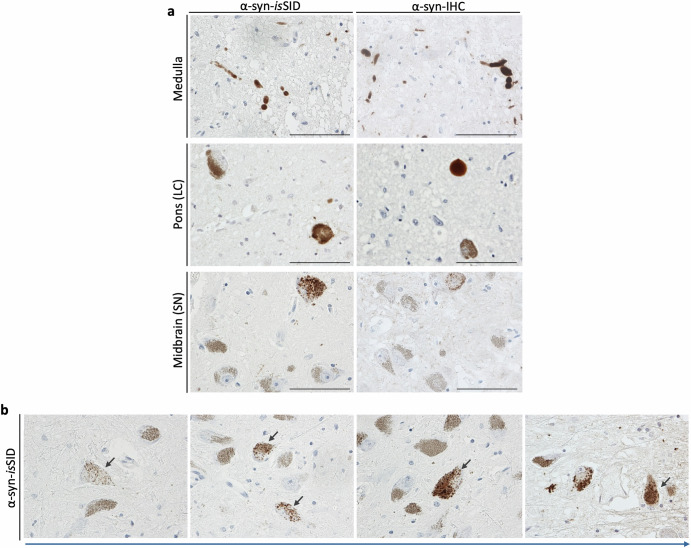
Seeding activity of α-syn is detected in the brainstem of incidental cases with LB-pathology. Incidental cases with LB-pathology but no clinical symptomatology had seeding-competent α-syn pathology as shown by α-syn-*is*SID. Pathology detected by α-syn-IHC in adjacent tissue sections is also shown (**a**). Proposed model of seeding and maturation stage of α-syn seeding in neuromelanin-positive neurons (arrows). Morphologically, the seeding activity of α-syn is observed first in a punctate pattern, which progressively increments in size and number until covering the whole neuronal cytoplasm and eventually forming LBs (**b**). Scale bar is 100 µm.

Consistent with their Braak stage, α-syn seeding activity was detected in neuronal inclusions within the medulla, pons, and midbrain (particularly in the LC and SN) (Fig. 8a). Oligodendrocytic α-syn seeding was scarce, and no α-syn seeding activity was observed in astrocytes. Interestingly, α-syn-*is*SID signal was detected in neuromelanin-containing dopaminergic neurons of the SN, indicating that these neurons harbor seeding-competent α-syn species ranging in the form of sparse punctate patterns to more condensed cytoplasmic structures (Fig. 8a). These distinct labeling patterns resemble the proposed stages of Lewy body morphogenesis, suggesting a temporal progression of α-syn aggregation^43,44^. Early punctate foci likely reflect the initial seeding events, which appear to increase in number and size over time, eventually occupying most of the cytoplasm and coalescing into mature LBs (Fig. 8b).

## Discussion

Neurodegenerative proteinopathies are characterized by the ability of misfolded aggregated proteins to seed the conversion of endogenous proteins into their pathological counterparts. However, current methodologies that detect seeding activity of biological samples lack spatial and cytoarchitectural resolution. Here, we describe our novel *is*SID assay for α-syn and tau proteins that overcomes these limitations. This approach enables the direct visualization of α-syn and tau seeding activities in situ, providing detailed insights into their spatial and cellular distribution.

Growing evidence supports the central role of protein aggregation and seeding in the pathogenesis of neurodegenerative proteinopathies. However, the seeding activity across distinct cell types and inclusion subtypes has remained elusive, particularly amid ongoing debate surrounding whether LBs exert a pathogenic or protective role in disease progression^45,46^. Here, we provide direct visual confirmation that the neuropathological hallmarks of PD/DLB (LBs), MSA (GCIs), and AD (NFTs) have biologically active seeding capacity. Beyond these canonical lesions, we also demonstrate that less common types of pathology, such as glial inclusions in PD as well as neuronal inclusions (NCIs and NNIs) in MSA, possess intrinsic seeding ability. Importantly, our findings confirm that LBs are neither inert nor dormant structures. Despite their compact and insoluble structure, they retain the ability to promote pathological protein aggregation, underscoring their active role in disease propagation. We observed striking heterogeneity in α-syn seeding activity at the single-cell level. Neurons completely lacking seeding-competent α-syn were frequently located adjacent to heavily burdened neurons, suggesting that α-syn propagation likely follows defined anatomical routes rather than occurring via passive diffusion^40,47,48^. Moreover, small seeding activity foci were also seen in the cytoplasm of a proportion of neurons and oligodendrocytes in PD and MSA, respectively. These punctate structures, including previously unreported “punctate-like GCIs”, may represent early intermediates in the formation of mature inclusions such as LBs and GCIs^49^. However, we cannot ascertain what proportion of neurons and oligodendrocytes harboring these punctate seeding foci will mature into fully developed inclusions or will undergo cell death^49^. We observed increasingly confluent small foci of α-syn seeding activity in the soma of a proportion of monoaminergic neurons in the SN and in the LC, which supports the proposed cellular maturation model. Given that monoaminergic neurons in the SN and in the LC are among the most selectively vulnerable neuronal populations, these neurons might have intrinsic properties predisposing them to α-syn seeding, even at early disease stages, ultimately contributing to their progressive dysfunction and death. In addition, we identified dot-like foci of α-syn seeding activity scattered in the neuropil which partially co-localize with presynaptic and neurofilament markers, suggesting that at least a proportion of them reside in neuronal projections and synapses^43,44,50^. This observation aligns with the proposed role of toxic oligomers at synapses and the potential transsynaptic spread of aggregates. Moreover, α-syn-*is*SID signal was observed surrounding neuritic plaques with a morphology indicative of an association with dystrophic neurites^48,49^. Notably, the non-Aβ component of the AD amyloid (NAC), a fragment of α-syn has been reported as an integral component of Aβ plaques^51^. The observed seeding activity of α-syn alone may be sufficient to seed full-length α-syn, in line with in vitro findings^52^. Nevertheless, the possibility of α-syn and Aβ or tau cross-seeding cannot be completely excluded, and further research directly investigating this is warranted.

In vitro studies have demonstrated that glia can actively propagate pathology; however, we now provide the first direct evidence that endogenous glial inclusions possess seeding capacity. These findings challenge the entirely neuron-centric view of PD by highlighting glia as active contributors to disease progression rather than passive bystanders. Recently, we have described different morphologies of astrocytic α-syn pathology in PD, which correlated with the density of astrocytic α-syn pathology, and here we show that all proposed morphologies of astrocytic pathology exhibit seeding activity^4^. Furthermore, our observation of α-syn-*is*SID signal present in microglia could support their involvement in disease dissemination^53^ but, whether seeding-competent glia are actively promoting pathology or if these represent sequestered toxic α-syn aggregates as a neuroprotective measure remains to be understood. The time required to reach the detection threshold during the lag phase is commonly used as a surrogate measure of seeding activity and seed concentration^54,55^. Hence, to investigate differential seeding activity between neuronal and glial α-syn pathology, we conducted the α-syn-*is*SID assay using very short incubation times. At the earliest timepoints, we observed a higher prevalence of neuronal compared to glial inclusions, suggesting a higher α-syn seed concentration within neuronal pathology or that neuronal-derived seeds might have a more potent seeding activity. Additionally, we detected more α-syn seeding-competent neurons than neuronal perikaryal lesions detectable by α-syn-IHC. These results suggest that morphologically intact neurons can harbor active seeding foci not detectable by α-syn-IHC, perhaps due to their small size. It could be speculated that these early seeding-competent forms may relate to the early aggregates we previously reported using AS-PLA^56^. However, the sensitivity and dynamic range of *is*SID in accurately detecting such small oligomeric seeds remain to be fully explored, particularly given the unknown concentration of seeds in the human brain. Moreover, iLBD is considered a pathological precursor of PD^57^, and our detection of α-syn seeding in iLBD cases confirms that seeding is already present at the presymptomatic stages^58^. This aligns with studies demonstrating that prodromal cases, such as those with REM sleep behavior disorder (RBD) or pure autonomic failure (PAF), are able to seed in SAAs despite lacking overt pathology^59–61^. Furthermore, our finding that neuronal α-syn seeding vastly predominates in iLBD cases, with very rare α-syn-*is*SID positive oligodendrocytic α-syn inclusions and no detectable astrocytic α-syn seeding, further supports the notion that neurons are most likely the main drivers of α-syn seeding. We recently reported that neuronal α-syn pathology appears first, followed closely by oligodendrocytic α-syn pathology, whereas astrocytic α-syn pathology emerges once neuronal and oligodendrocytic pathology is established. In this study, we confirm that this neuroanatomical pattern of progression is also reflected at the seeding level, reinforcing the concept that α-syn spread follows a defined, hierarchical cellular trajectory.

While in this study we mostly focused on α-syn, we hypothesized that our method could be applied to other proteinopathies, such as tauopathies. In addition, we wondered if the endogenous neuronal and glial α-syn seeding revealed in PD and MSA cases was parallel to tau seeding activity in AD. Using tau-*is*SID, we found, in contrast to α-syn, that tau seeding activity in AD seemed to be mostly restricted to neuronal elements, including mature NFTs and neuropil threads, as well as prominent dot-like seeding activity foci scattered in the neuropil. However, tau seeding activity needs to be further explored in a larger cohort, including other tauopathies such as Progressive Supranuclear Palsy, which is characterized by tau pathology in glia and a distinct neuroanatomical involvement.

Neurodegenerative diseases are becoming increasingly prevalent, with key challenges including the lack of early diagnostic methods and effective treatments. Early diagnosis is crucial for the identification of novel biomarkers, the development of new therapeutics, and the advancement of personalized medicine. By applying the *is*SID assay, we have uncovered previously inaccessible data, providing novel insights into the mechanisms underlying the propagation of pathological aggregates and offering potential applications for the development of diagnostic tools. Nevertheless, the functional link and biological significance of seeding activity in relation to neurodegeneration, synaptic dysfunction, or clinical measures remain elusive, along with the question of whether the detected *is*SID-positive seeds are biologically active, transmissible, or toxic. This approach holds further potential application in in vitro models, offering insights into seeding mechanisms at the subcellular level. For example, in combination with electron microscopy, this assay could identify the organelles and cellular structures involved in protein aggregation. Similarly, the *is*SID assay may serve as a valuable tool for studying maturation of pathology in animal models, enabling investigations into whether punctate seeding foci evolve into mature α-syn inclusions. Moreover, in vitro systems could be leveraged to evaluate the efficacy of therapeutic compounds targeting pathological seeding. A similar rationale can be applied at the subcellular level. If seeding activity is shown to be predominantly associated with specific organelles, targeted therapeutic strategies could be developed to selectively disrupt those compartments. Additionally, the integration of immunodetection and His-tagged monomers could enhance the specificity and sensitivity of RT-QuIC performed on bulk tissue, enabling the delineation of newly formed aggregates (containing the His-tag) in combination with other analytical techniques. Importantly, we have successfully shown that the novel *is*SID assay can be adapted to understand the cellular and spatial aspects of seeding in other proteinopathies. It could be expanded further to include, for example, Aβ and TDP-43, as well as mutant protein substrates and specific tau isoforms. To this respect, while shaking is most likely required to enhance tau-*is*SID, it did not significantly enhance α-syn-*is*SID. Future studies aiming to adapt the *is*SID assay for other proteinopathies will likely require a flexible platform to allow the optimization of the incubation parameters, such as buffer composition, protein concentration, shaking and temperature.

The adaptation of *is*SID for use with tissue sections marks a significant advancement. FFPE tissue is widely available and often archived, allowing for retrospective analyses of well-characterized cases. While we acknowledge that the seeding capacity observed in homogenized frozen tissue may not be fully recapitulated in FFPE tissue, our results, particularly in distinguishing positivity or negativity, align with results using bulk SAAs. Similarly, we cannot exclude the possibility of selective amplification bias, as experimental conditions may preferentially amplify certain α-syn species, such as α-syn strains, as observed in bulk seeding assays^62–64^. Another important consideration is the variability in fixation times and tissue processing protocols across brain banks. Prolonged fixation can lead to protein degradation or reduced extractability, potentially introducing inconsistencies in seeding activity. Nonetheless, we did not observe any correlation with *post-mortem* interval or fixation times. Additionally, as the *is*SID assay uses immunodetection such as IHC, which relies on signal amplification, the size of the observed seeding foci may not directly reflect their true dimensions in situ. Comparisons between α-syn seeding foci detected by our novel assay and those identified by IHC could help clarify this discrepancy. Further differences in incubation times and antibody concentrations may also influence the signal developed by both assays. Although we demonstrated that cases with a positive RT-QuIC response also exhibit a positive *is*SID signal, it remains to be determined whether the load of seeding-competent lesions detected by *is*SID directly correlates with kinetic parameters of bulk RT-QuIC, as well as their relative specificity and sensitivity, such as whether a single seeding-competent lesion identified by *is*SID would produce a visible increase in bulk ThT fluorescence.

In conclusion, the novel *is*SID assay described here enables streamlined visualization of seeding activity with unprecedented spatial and cellular resolution. This significant advancement provides valuable insight into the molecular underpinnings of disease pathogenesis and progression in neurodegenerative disorders, facilitating a deeper understanding of the dynamics of protein aggregation and misfolding.

## Methods

### *Post-mortem* human tissue

The cohort was composed of PD (*n* = 24), MSA (*n* = 12), DLB (*n* = 11), iLBD (*n* = 8), AD (*n* = 3), and healthy controls (*n* = 6). FFPE and snap-frozen *post-mortem* human tissue were obtained from The Multiple Sclerosis and Parkinson’s UK Brain Bank at Imperial College London and used under Research Ethics Committee Approval Ref. No 23/WA/0273. Regions analyzed included the amygdala, midbrain, pons, medulla, cerebellum, and hippocampus, as well as the superior frontal, anterior cingulate, temporal, and entorhinal cortices. Detailed cohort information can be found in Supplementary Table 1.

### *Is*SID on tissue sections

This technique has a filed patent, application number GB2410222.0. For α-syn-*is*SID, FFPE-tissue sections were sectioned with a microtome at a thickness of 5 µm and baked at 55 °C for 45 minutes prior to dewaxing in xylene and rehydration in sequential concentrations of ethanol. The endogenous peroxidase activity was blocked in 0.3% H_2_O_2_ for 30 minutes at room temperature (RT), and antigen retrieval was performed in citrate buffer, pH 6.0, for a total microwave heating of 10 minutes. Slides were washed with phosphate-buffered saline (PBS) and preconditioned with the buffer of choice (PBS, 80 mM NaCl (pH 7.4), or PIPES buffer (pH 6.9) for 1 hour. For the *is*SID under shaking conditions, cylinders were placed on a thin layer of low viscosity silicone grease (Sigma, 769711). The cylinder was placed in the region of interest (ROI), and the reaction buffer was added to the cylinder. The buffer of α-syn*-is*SID contained 0.025 mg/ml of recombinant WT human His-tagged α-syn (His-α-syn, Bio-techne, SP-480) or A53T His-α-syn (Sigma, S1071), the designated buffer and 10 µM ThT. Unless specified, WT human His-α-syn was used throughout the study. To maintain consistent humidity during slide incubation, we designed and developed prototypes, including a plate adapter, roofed chambers with a silicone foam gasket, and cylinders with thick walls (Supplementary Fig. 7). The slides were placed into the plate holder and sealed with roofed chambers before incubating in a FLUOstar Omega microplate reader (BMG Labtech), which underwent orbital shaking at 500 rpm for 1 minute every 15 minutes at 37 °C. Under non-shaking conditions, hydrophobic PAP was used to retain the reaction buffer on the tissue sections. Following incubation, the roofed chambers were removed, and 200 µL of 4% paraformaldehyde (PFA) was added to the cylinder prior to its careful removal and incubated for 10 minutes (optional). Subsequently, the tissue sections were washed with 0.5% PBS-Tween and blocked for 1 hour with 10% normal horse serum (NHS). The sections were then incubated with a 1:5000 dilution of anti-His-tag antibody for 2 hours at RT. Sections were visualized with ImmPRESS[R] HRP Horse Anti-Rabbit IgG Polymer Detection Kit (Peroxidase) and ImmPact DAB Substrate HRP. Tissue sections were counterstained with haematoxylin, dehydrated with ethanol and xylene prior to coverslipping with Epredia™ ClearVue™ Coverslipper. The workflow of the *is*SID assay is represented in Fig. 1.

The same procedure was followed for immunofluorescence-based *is*SID assay but omitting the ThT from the reaction buffer. Tissue sections were blocked for 1 hour with 10% normal donkey serum (NDS) and incubated with the primary antibodies for 4 hours at RT. Slides were incubated with 0.1% Sudan Black before mounting with VECTASHIELD® Antifade Mounting Medium with DAPI. The antibodies and the concentrations used are listed in Supplementary Table 2.

For tau-*is*SID, the same protocol was followed but with key modifications in the reaction buffer composition and incubation conditions. The reaction buffer contained 10 mM HEPES (pH 7.4), 400 mM NaCl, 40 µM heparin, 10 µM ThT, and 0.25 mg/mL His-tagged τ306 tau fragment (His-tau), which encompasses residues 306–378 of the full-length human tau isoform (htau40), with a C322S mutation and a stop codon at residue 379^65^. Tau-seeded slides were incubated at 42 °C under orbital shaking at 500 rpm for 1 minute on/1 minute off. Post-incubation steps remained unchanged, except for incubation with a 1:500 dilution of anti-His-tag antibody (Fig. 1).

### *Is*SID on pre-formed fibrils

PFFs of α-syn were made as detailed above and were serially diluted from starting concentrations of 1 mg/mL down to 1 µg/mL. Diluted PFFs were spotted onto poly-D-lysine-coated coverslips and fixed with 4% PFA for 30 minutes at RT. The *is*SID assay was then carried out as described above. Control conditions included monomers as well as PFFs incubated without His-tagged protein. The His-tag signal was detected using fluorescently labeled secondary antibodies, and the coverslips were mounted for imaging. Immunofluorescence on the α-syn PFFs was performed with anti-α-syn antibody clone 42.

### Transmission electron microscopy

Transmission electron microscopy was performed by adding 10 µL of the sample to pre-glow discharged carbon formvar copper grids for 2 minutes. Grids were blotted and transferred to 2% aqueous uranyl acetate droplet for 1 minute and then transferred to filtered Milli-Q water for 1 minute. Grids were blotted and dried again. FEI Tecnai 12 TEM (120 kV) was used for imaging and images taken with Gatan US1000 camera.

### Immunohistochemistry

FFPE-tissue sections were dewaxed and rehydrated as described above. The endogenous peroxidase activity was blocked in 0.3% H_2_O_2_ for 30 minutes at RT, and antigen retrieval was performed in 100% formic acid for 10 minutes. The tissue sections were blocked in 10% NHS and incubated with the primary antibody overnight at 4 °C. Next day, the slides were incubated with ImmPRESS[R] HRP Horse Anti-Mouse IgG Polymer Detection Kit for 30 minutes at RT. ImmPact DAB Substrate HRP was added, and sections were counterstained with haematoxylin, dehydrated with ethanol and xylene, before being coverslipped using Epredia™ ClearVue™ Coverslipper. The antibodies and the concentrations used are listed in Supplementary Table 2.

### AS-PLA/tau-PLA

Both α-syn and tau Proximity Ligation Assay (AS-PLA/tau-PLA)^56,66^ conjugates were generated by conjugating either anti-α-syn antibody MJFR1 (Abcam, ab209420) or anti-tau antibody TAU-5 (Abcam, ab80579) to PLA oligonucleotides using the Duolink® In Situ Probemaker PLUS (Sigma, DUO92009) and MINUS (Sigma, DUO92010) kits^56,66^. FFPE-tissue sections were deparaffinized and rehydrated as previously described. Endogenous peroxidase activity was blocked in 10% H_2_O_2_ at RT for 1 hour, followed by heat-mediated antigen retrieval in the microwave. Tissue sections were incubated with endogenous avidin and biotin blocking solution (Biolegend, 927301) as per manufacturer’s instructions before incubating with the provided PLA blocking solution for 1 hour at 37 °C. Subsequently, PLA conjugates were diluted in the Probemaker diluent and incubated overnight at 4 °C. The following day, the assay was performed as described in Söderberg et al.^67^ with minor modifications. Sections were incubated with T4 ligase buffer, two connector oligonucleotide probes, adenosine triphosphate (ATP), and T4 ligase for 1 hour at 37 °C. After washing with tris-buffered saline (TBS) 0.05% Tween, rolling circle amplification was performed by adding the phi29 polymerase buffer, deoxynucleotide triphosphates (dNTPs), and phi29 polymerase, followed by incubation for 2.5 hours at 37 °C. Detection was then carried out by adding detection buffer containing saline-sodium citrate (SSC), polyadenylic acid (poly-A), bovine serum albumin (BSA), 0.5% Tween, and biotin-labeled oligonucleotide, followed by incubation for 1 hour at RT. Subsequently, the slides were incubated with Strepavidin-HRP solution (Abcam, AB64269) and developed with Vector NovaRed substrate. Finally, the sections were counterstained, dehydrated, and coverslipped using the Epredia™ ClearVue™ Coverslipper.

### Image analysis

Brightfield images were acquired with a DS-Fi2 Digital Camera attached to a Nikon Eclipse 50i microscope, using a DS-U3 Digital Camera Controller and NIS-Elements image acquisition software. Immunofluorescence slides were imaged at 40x magnification with a numerical aperture of 0.95 on an Olympus BX63 scanning fluorescence microscope implementing the cellSens imaging software.

### RT-QuIC

The RT-QuIC assay was performed according to the previously described methods^17,45^. First, snap-frozen tissue samples were homogenized in ice-cold PBS (10% w/v) using a Tissue Ruptor II with disposable probes (Qiagen). For α-syn and tau RT-QuIC, brain homogenates were diluted to a final concentration of 1 × 10^–3^ and 1 × 10^–1^, respectively. Undiluted α-syn PFFs were added to α-syn RT-QuIC.

Using slightly modified reaction mixtures as for *is*SID, reactions were performed in triplicate in a black 96-well plate with a clear bottom (Nunc, Thermo Fischer). A total of 98 µL of reaction mix was loaded into each well along with 2 µL of diluted brain homogenate. The plate was sealed with a sealing film (Thermo Fisher) and incubated in a BMG Omega FLUOstar OMEGA plate reader at 42 °C for 90 hours, with intermittent double orbital shaking at 500 rpm for 15 minutes throughout the incubation time. The assay cut-off was established at 70 hours, representing a reproducible endpoint prior to the onset of spontaneous amyloid aggregation in the wells.

### Quantification of cell-specific α-syn pathology

The classification of cell-specific α-syn pathology was performed as previously described^4^. In brief, stained tissue sections were scanned using the Leica Aperio AT2 slide scanner. QuPath software (version 0.4.4.)^68^ and StarDist (version 0.3.2.), a deep learning approach^69^, were used for the development of artificial neural network (ANN)-classifiers to assist in the supervised detection of cell type-specific α-syn inclusions. Cells that were detected by StarDist and QuPath were used to train ANN-classifiers by manual annotation. After training the ANN-classifiers, three ROIs corresponding to a 20x field of view were drawn, and the ANN-classifiers were applied. To determine the average percentage area covered by pathology, ROIs were extracted to FIJI (version 2.3.0.) and underwent color deconvolution to isolate the chromogen channel. The images were thresholded, and the average fraction of the total positive area was calculated.

### Statistical analysis

GraphPad Prism software was used for statistical analysis. The levels of α-syn pathology in neurons, astrocytes, and oligodendrocytes, as detected by IHC and *is*SID, were compared by two-way ANOVA followed by Bonferroni’s *post-hoc* test. Statistical significance was set at *p* < 0.05. Spearman’s rank correlation was used to assess correlation relationships between percentage area detected by two techniques, with the Spearman *r* and *p* values reported.

## Supplementary information

Supplementary information

## Data Availability

All data are available in the main text or Supplementary Materials. The raw data that support the findings of this study are available from the corresponding author upon request.
