# Supplementary material for: Novel in situ seeding immunodetection assay uncovers neuronal-driven alpha-synuclein seeding in Parkinson’s disease
Source: NPJ Parkinsons Dis. 2025 Aug 25;11:259. doi: 10.1038/s41531-025-01111-y (PMC12379234; doi:10.1038/s41531-025-01111-y)
Supplement: Supplementary file 1 — Supplementary information [file 41531_2025_1111_MOESM1_ESM.pdf]

Inventory of Supporting Information

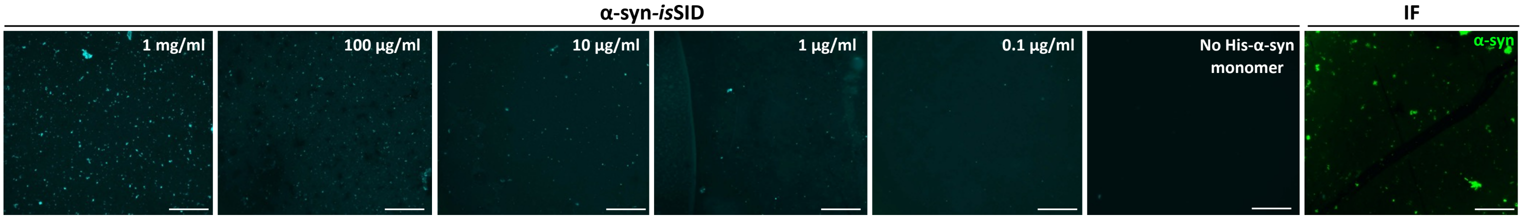

Supplementary Fig. 1: Sensitivity validation of the α-syn-*is*SID assay. The α-syn-*is*SID assay was performed on serial dilutions of α-syn PFFs spotted onto poly-D-lysine-coated coverslips. A robust fluorescent signal was detected at 1 mg/mL, with progressively reduced signal across dilutions. Notably, signal remained detectable at 1 μg/mL, indicating the assay's sensitivity. Immunofluorescence (IF) with α-syn antibody (clone 42) confirmed the presence of α-syn PFFs. Scale bar is 100 μm.

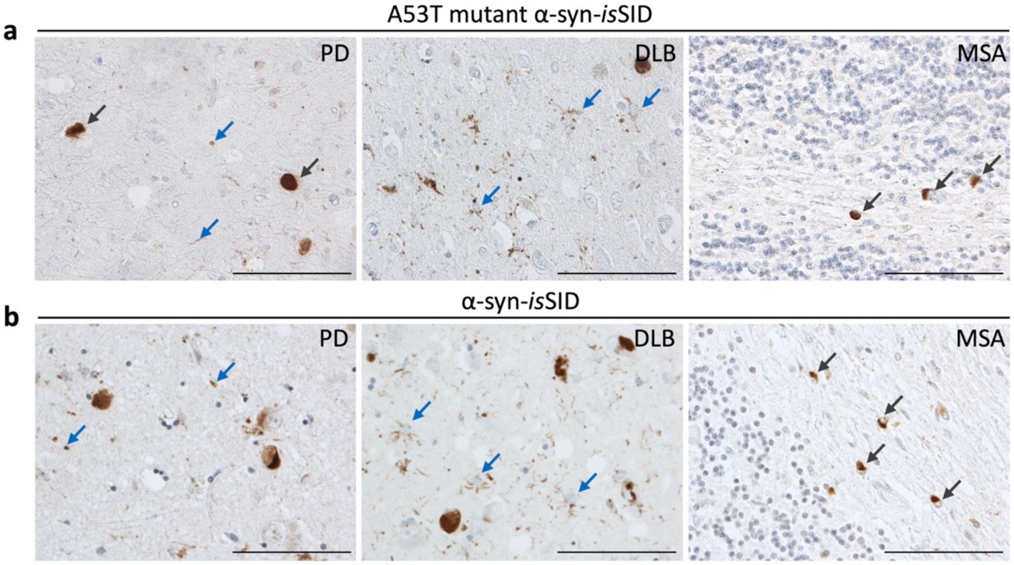

Supplementary Fig. 2: α-Syn-*is*SID using A53T mutant α-syn protein in PD, DLB, and MSA cases. LB (grey arrow) and dot-like/neuritic pathology (blue arrow) were seen in PD. In DLB cases, astrocytic α-syn pathology is depicted (blue arrows). GCIs were detected in MSA (grey arrows) (**a**). A comparison of results with α-syn-*is*SID (using WT human his-α-syn as throughout the manuscript) is shown (**b**). Scale bar is 100 μm.

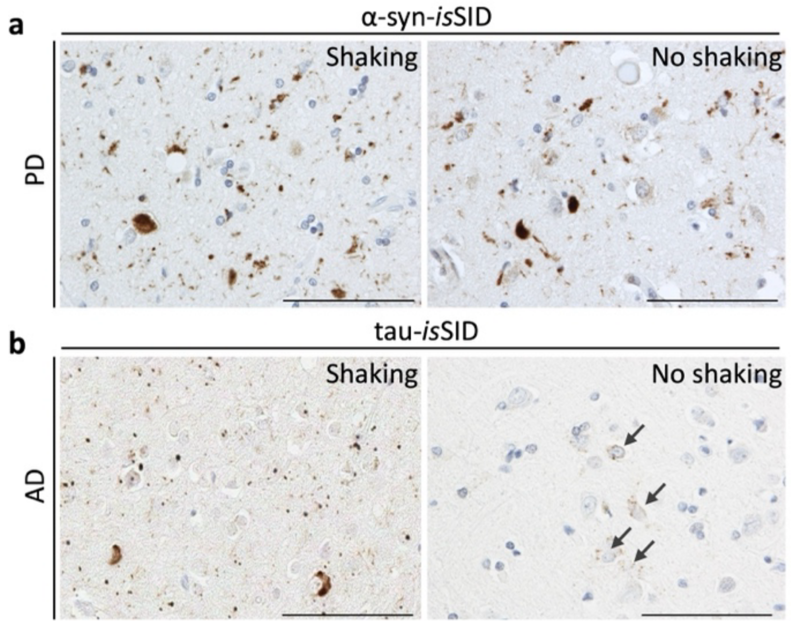

<u>Supplementary Fig. 3</u>: Representative images with shaking on α-syn-*is*SID and tau-*is*SID assays. Shaking had no impact on α-syn-*is*SID signal (**a**). In contrast, tau-*is*SID signal was lower under non-shaking conditions, although dot-like neuropil and punctate cytoplasmic staining remained detectable (**b**). Scale bar is 100 μm.

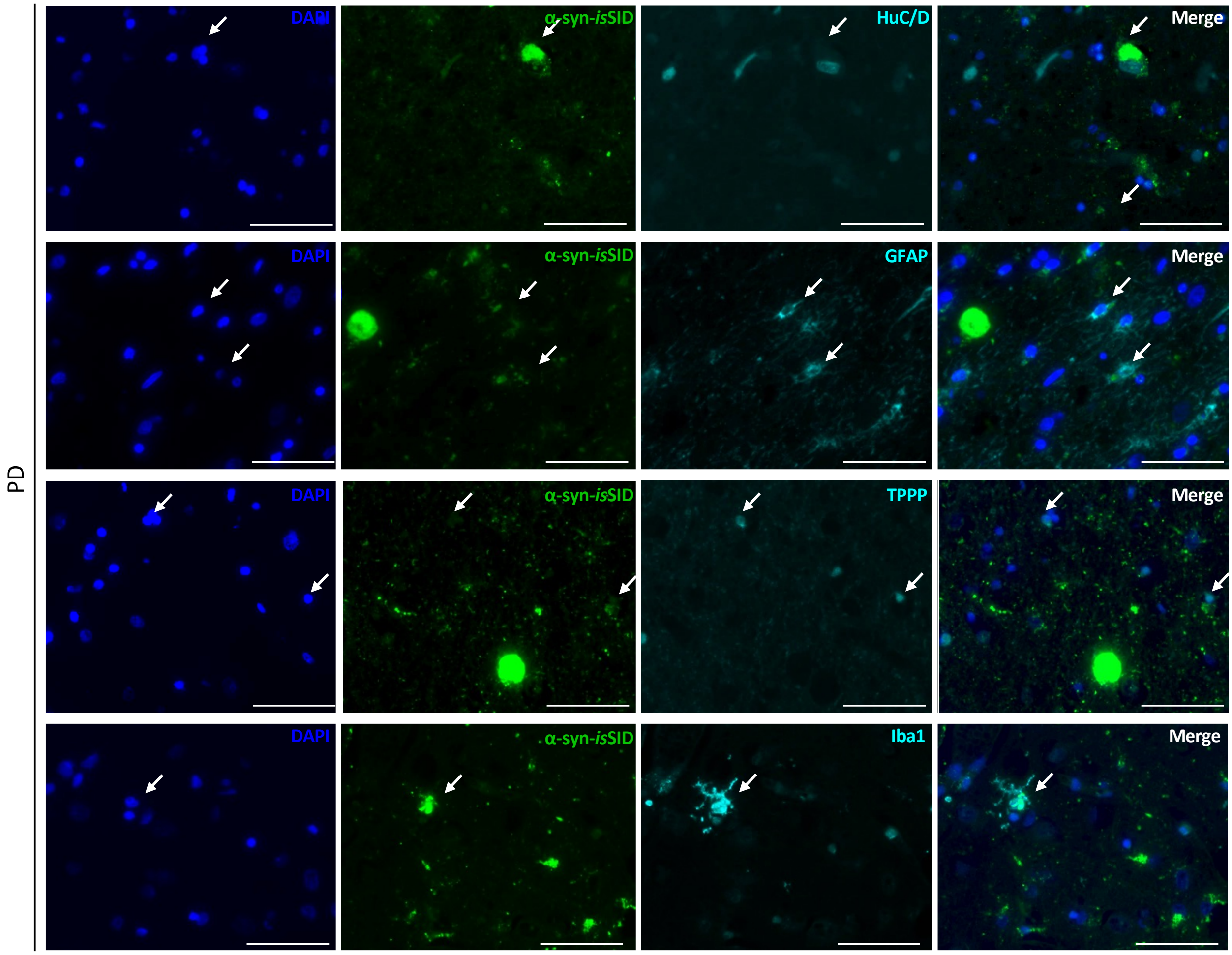

<u>Supplementary Fig. 4</u>: **Immunofluorescence confirmed that α-syn seeding activity is present within neurons, astrocytes, oligodendrocytes, and microglial α-syn.** Co-localization of α-syn-*is*SID signal with cell-type-specific markers, HuC/D (neurons), GFAP (astrocytes), TPPP (oligodendrocytes), and Iba1 (microglia) demonstrates the seeding capacity of α-syn inclusions in these cell types. DAPI was used as a nuclear marker. Arrows depict areas of co-localization. Scale bar is 100 µm.

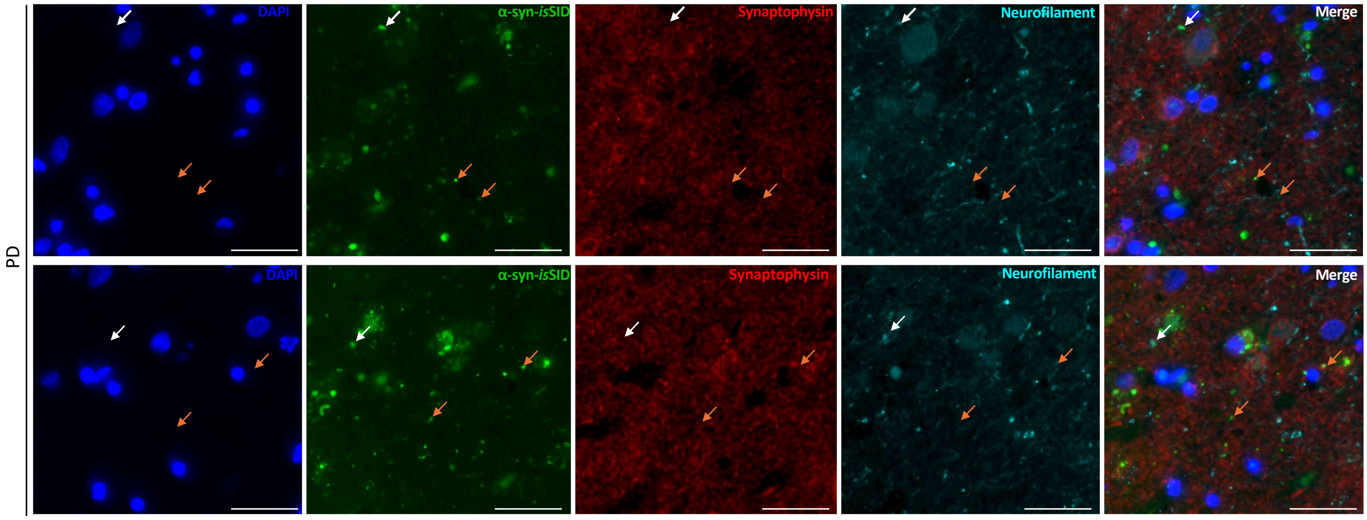

<u>Supplementary Fig. 5</u>: Immunofluorescence shows dot-like pathology in the synapses and in close proximity. Dot-like α-syn-*is*SID signal co-localized with pre-synaptic markers (synaptophysin) and neuronal marker (neurofilament heavy), being observed proximal to the synapses. Arrows depict areas of co-localization with synaptophysin (orange) and neurofilament heavy (white). Scale bar is 100 µm.

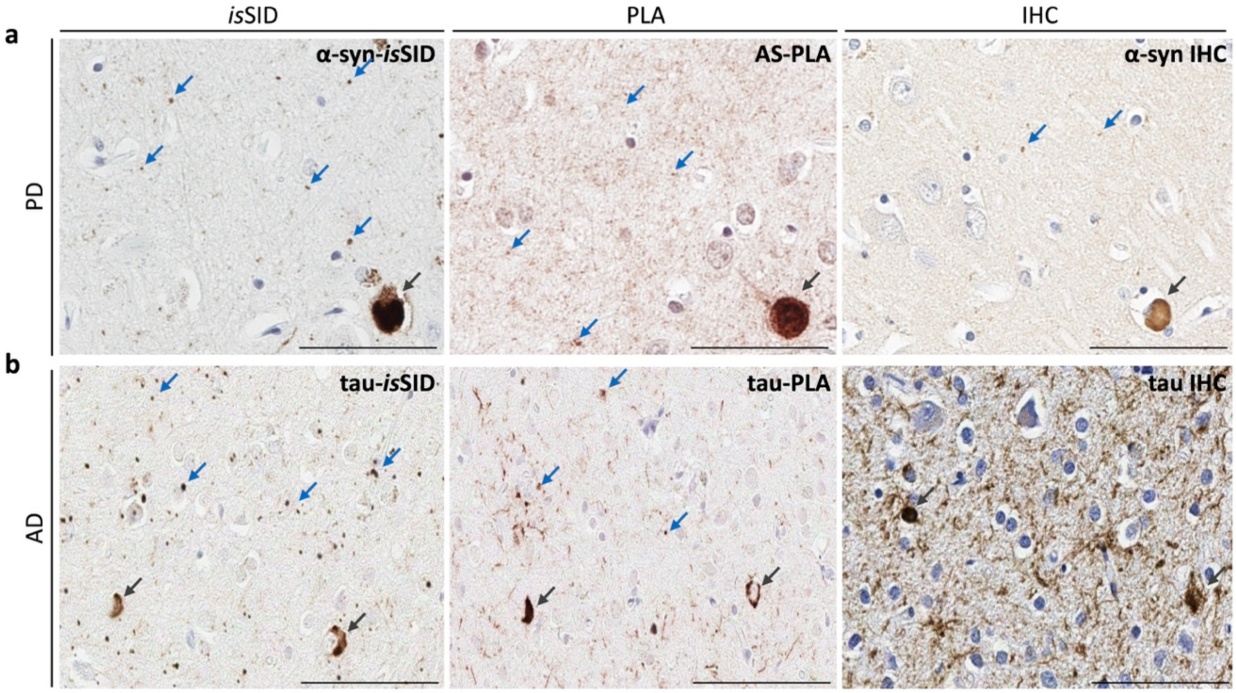

<u>Supplementary Fig. 6</u>: Comparison of signal detected by α-syn or tau *is*SID, PLA, and IHC. α-Syn aggregates, such as LBs, were detected by α-syn-*is*SID, AS-PLA, and α-syn IHC. However, dot-like α-syn pathology revealed by α-syn-*is*SID was more readily detected by AS-PLA than α-syn-IHC in PD cases (**a**). Similarly, tau aggregates were detected by all tau-*is*SID, tau-PLA, and tau-IHC. Notably, tau dot-like pathology revealed by tau-*is*SID closely overlapped with tau-PLA signal (**b**). Scale bar is 100 µm.

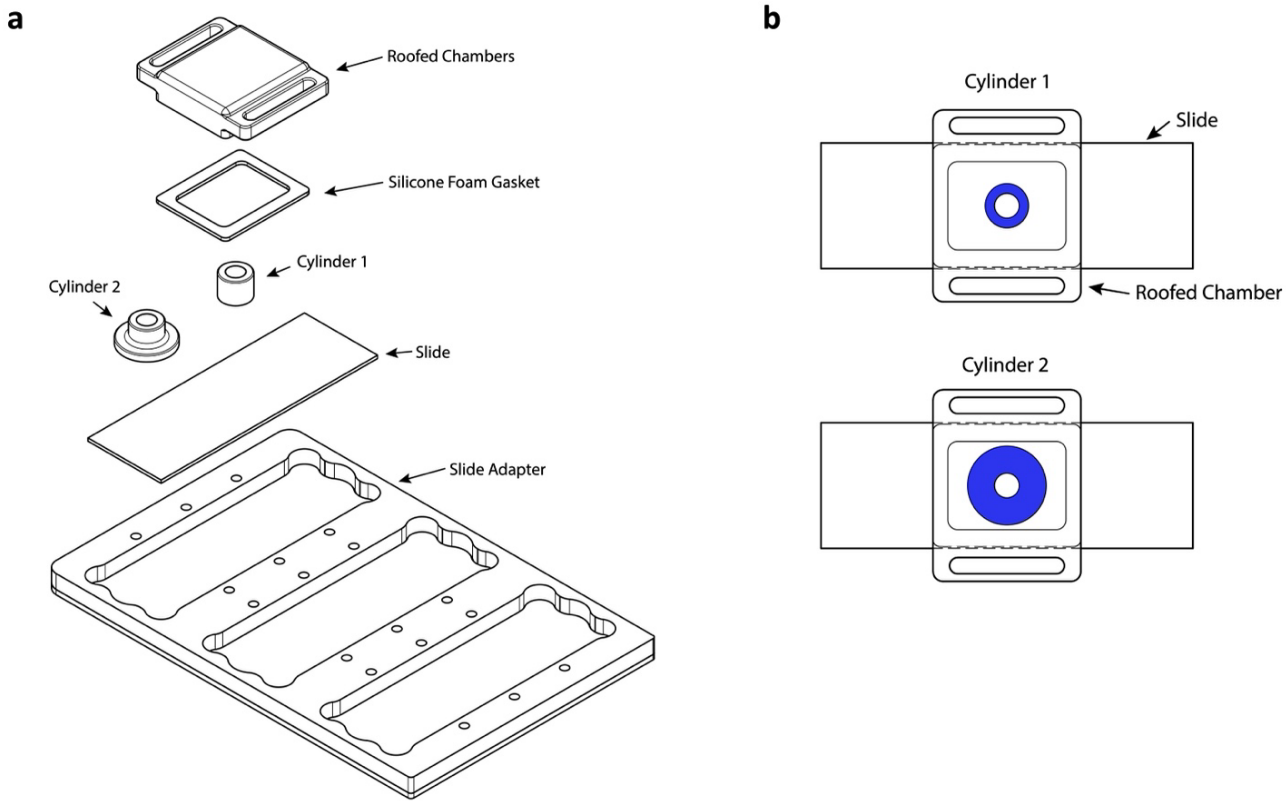

<u>Supplementary Fig. 7</u>: Newly designed prototype plate adapter and cylinder for the *is*SID incubation performed in the plate reader. A plate adapter with the dimensions of a 96-well plate was used to hold the slides during incubation. Then, the cylinder was stuck onto the tissue area of interest and filled with the reaction buffer. The roofed chambers, composed of a roof and a silicone foam gasket, secured the slides and cylinders during incubation by preventing liquid evaporation and leakage (**a**). The schematic diagram represents the view of the assay set up from the top. The cylinder consisting of thin walls (1) was redesigned into a new cylinder with thicker walls (2), which covered most of the tissue section being assayed (**b**).

Disease	Gender	Age at death	Disease duration	α-syn (Braak)	Tau (Braak)	Beta-amyloid (Thal)
MSA	M	80	11	n/a	I	0
MSA	F	85	9	n/a	V	3
MSA	F	77	8	n/a	IV	3
MSA	F	67	8	n/a	0	3
MSA	M	66	4	n/a	II	1
MSA	F	49	27	n/a	I	0
MSA	M	71	14	n/a	III	3
MSA	F	68	4	n/a	0	2
MSA	F	91	5	n/a	0	2
MSA	F	70	5	n/a	II	2
MSA	F	65	8	n/a	I	0
DLB	M	84	6	6	I	
DLB	M	58	15	6	I	2
DLB	M	63	9	6	IV	2
DLB	F	82	5	6	III	3
DLB	F	76	5	6	V	3
DLB	M	77	3	6	II	3
DLB	M	70	11	6	V	3
DLB	M	89	5	6	VI	3
DLB	M	80	5	6	VI	3
PDD/DLB	F	74	11	6	VI	5
PDD/DLB	F	84	12	6	IV	4
PD	F	90	22	3	II	0
PD	F	66	8	3	II	1
PD	M	75	19	3	II	2
PD	M	87	9	3	II	1
PD	M	73	7	3	I	3
PD	M	68	24	3	0	3
PD	F	75	9	4	III	5
PD4	M	86	13	4	III	3
PD	M	77	1	4	II	3
PD	M	76	16	4	0	0
PD	M	64	7	4	I	0
PD	M	76	13	4	I	0
PD	M	81	13	4	II	I
PD	F	84	13	5	III	3
PD	F	84	16	5	II	1
PD	M	75	12	5	II	2
PD	M	80	20	5	II	2

PD	M	88	10	5	I	0
PD	M	87	20	5	III	3
PD	M	72	4	5	I	2
PD	F	83	15	6	IV	5
PD	M	73	22	6	II	0
PD	M	87	10	6	I	0
PD	F	82	21	6	III	4
Control	F	61	n/a	0	I	0
Control	F	60	n/a	0	I	0
Control	M	54	n/a	0	0	0
Control	F	63	n/a	0	I	0
Control	M	32	n/a	0		
Control	F	50	n/a	0	I	0
ILBD	F	82	n/a	1	II	2
ILBD	F	98	n/a	2	III	3
ILBD	M	88	n/a	AMY	IV	4
ILBD	F	86	n/a	3		
ILBD	M	91	n/a	1	I	3
ILBD	F	77	n/a	3	II	3
ILBD/AD	F	98		3	VI	
ILBD/PSP	F	93	13	1	II	3
AD	M	77	8	0	IV	3
AD	M	79	n/a	0	VI	5
AD	F	72	n/a	0	V	4

Supplementary Table 1: Detailed cohort information. Disease, gender, age at death, disease duration, α-syn (Braak staging), tau (Braak staging), and amyloid-beta (Thal phases) are reported.

**Antibodies/Fluorophores**	**Species**	**Catalogue number**	**RRID**	**Concentration**

His tag (Thermofisher)	Rabbit	RM146	AB_2810125	1:500 - 1:5000 IHC / 1:500 IF
Clone 42 (BD Biosciences)	Mouse	610787	AB_398107	1:1000 IHC /1:400 IF
Synaptophysin (Biolegend)	Mouse	837104	AB_2783411	1:100 IF
PSD95 (Biolegend)	Mouse	810301	AB_2564749	1:100 IF
HUC/D (Thermofisher)	Mouse	A-21271	AB_221448	1:200 IF
GFAP (Thermofisher)	Rat	13-0300	AB_2532994	1:1000 IF
Neurofilament heavy chain (Sigma Aldrich)	Chicken	AB5539	AB_11212161	1:750 IF
Iba1 (Abcam)	Goat	AB5076	AB_91676	1:400 IF
TPPP (Thermofisher)	Goat	PA5-19243	AB_10979400	1:200 IF
Mjfx (Abcam, BSA and azide free)	Rabbit	AB209420	AB_2537217	1:200 PLA
Tau5 (Abcam, BSA and azide free)	Mouse	AB80579	AB_1603723	1:250 PLA
Donkey Anti-Rabbit (Alexa Fluor® 647) (Abcam)		AB150075		1:750 IF
Donkey Anti-Mouse(Alexa Fluor® 488) (Abcam)		AB150105		1:750 IF
Goat anti-mouse IgM (Alexa Fluor® 568) (Thermofisher)		A21043		1:750 IF
Donkey anti-Goat (Alexa Fluor® 568) (Thermofisher)		A11057		1:750 IF
Goat anti-chicken (Alexa Fluor® 647) (Thermofisher)		A21449		1:750 IF

Goat anti-mouse IgG2b (Alexa Fluor® 488) (Thermofisher)		A21141		1:750 IF
Goat anti-rat (Alexa Fluor® 568) (Thermofisher)		A11077		1:750 IF
Goat anti-rabbit (Alexa Fluor® 488) (Thermofisher)		A11034		1:750 IF
Goat anti-mouse IgG1 (Alexa Fluor® 647) (Thermofisher)		A21240		1:750 IF

Supplementary Table 2: Antibodies used in the study, including species, catalogue number, RRID, and concentration. The concentration used for immunohistochemistry (IHC), immunofluorescence (IF) and proximity ligation assay (PLA) is reported separately where applicable.
